# Case Report: Subacute cutaneous lupus erythematosus induced by the anti-PD-1 antibody camrelizumab combined with chemotherapy

**DOI:** 10.3389/fimmu.2025.1539373

**Published:** 2025-03-28

**Authors:** Hejing Bao, Jiani Zhang, Xi Luo, Xiaojing Song, Juan Li, Nan Mao, Fang Chen, Hehong Bao, Jiazhu Hu, Xiaolong Cao, Shudong Ma, Liping Lin

**Affiliations:** ^1^ Department of Oncology, The Affiliated Panyu Center Hospital, Guangzhou Medical University, Guangzhou, Guangdong, China; ^2^ Cancer Institute of Panyu District, The Affiliated Panyu Center Hospital, Guangzhou Medical University, Guangzhou, Guangdong, China; ^3^ Department of Oncology, Nanfang Hospital, Southern Medical University, Guangzhou, Guangdong, China; ^4^ Department of Dermatology, Shenzhen University General Hospital, Shenzhen, Guangdong, China; ^5^ Department of Pharmacy, The Affiliated Panyu Center Hospital, Guangzhou Medical University, Guangzhou, Guangdong, China; ^6^ Department of Pathology, The Affiliated Panyu Center Hospital, Guangzhou Medical University, Guangzhou, Guangdong, China; ^7^ Department of Psychosomatic Medicine, Chongqing University Three Gorges Hospital, Chongqing, Chongqing, China

**Keywords:** subacute cutaneous lupus erythematosus, non-small cell lung cancer, immune checkpoint inhibitors, camrelizumab, case report

## Abstract

The use of immune checkpoint inhibitors (ICI) can lead to immune-related adverse events (irAE), of which skin irAE is common, affecting up to 50% of ICI-treated patients. Although only a few cases of subacute cutaneous lupus erythematosus (SCLE) have been reported in patients receiving anti-programmed death-1(anti-PD-1) immunotherapy, it is important to identify ICI-induced SCLE because it may cause delayed and/or prolonged skin reactions even after treatment discontinuation. To date, no cases of cutaneous lupus associated with Camrelizumab treatment have been reported.

**Case report:**

We report a case of a patient with advanced non-small cell lung cancer (NSCLC) who gradually developed erythematous rashes on sun-exposed skin with pruritus after one course of anti-PD-1 antibody Camrelizumab combined with chemotherapy. The rashes were initially considered as eczema, but did not improve after symptomatic treatment. The rashes continued to worsen after the third course of treatment, and the pruritus was unbearable. After antibody testing, the patient was found to have positive anti-SS-A/Ro antibody, and the histological changes were consistent with subacute cutaneous lupus erythematosus. SCLE was controlled with local and systemic glucocorticoids, hydroxychloroquine, and discontinuation of anti-PD-1 therapy.

**Conclusion:**

Camrelizumab treatment may be associated with the appearance of subacute cutaneous lupus erythematosus in sun-exposed skin regions, which can be rapidly relieved by local and systemic glucocorticoids and hydroxychloroquine. It is recommended to perform early antibody testing and skin biopsy for diagnosis and treatment. Unlike classic drug-related SCLE, patients may develop multiple autoimmune diseases, and caution should be taken when using immune checkpoint inhibitors for subsequent treatment.

## Introduction

Patients with cancer who receive immune checkpoint inhibitors may experience skin immune-related adverse events (cirAEs) of various subtypes, including macules, pruritus, lichenoid, immune-mediated bullous, psoriasis-like, erythema multiforme or Stevens-Johnson syndrome, drug eruptions with eosinophilia and systemic symptoms, connective tissue diseases, etc. (1). Among them, macules, pruritus, psoriasis-like, and lichenoid rashes are the most common subtypes (2). Skin immune-related adverse events occur early, with macules appearing within the first 6 weeks of initial immune checkpoint inhibitor use (3, 4). Most skin AEs are low grade, with less than 3% progressing to grade 3 or 4 reactions (5). Camrelizumab is a humanized IgG4-κ monoclonal antibody targeting programmed cell death protein 1, showing anti-tumor activity and tolerability in various tumors, including lung cancer (6–13). In the phase III CameL-sq trial, the results showed that the combination of camrelizumab with chemotherapy significantly prolonged the progression-free survival (median, 8.5 months vs. 4.9 months; p<0.0001) and overall survival (median, not reached vs. 14.5 months; p<0.0001) compared with placebo plus chemotherapy (6). In lung cancer-related studies, the incidence of rashes with camrelizumab plus chemotherapy was 13%-18.6% and 5.3%-6.7%, respectively, while the incidence of rashes with placebo plus chemotherapy was 5.3%. Among them, the incidence of grade 3 or higher rashes with camrelizumab plus chemotherapy was 0-2.0%, while that with chemotherapy alone was 0% (1, 2, 14, 15).

Subacute cutaneous lupus erythematosus accounts for about 8% of all cutaneous lupus erythematosus (CLE) cases and lasts longer than acute lupus erythematosus (LE). It is highly photosensitive (16). It has a symmetrical distribution, most commonly found in the exposed neck area, upper trunk, and upper limbs, but usually not in the central facial area (17). It presents in two forms, both accompanied by erythematous plaques, one being a multi-annular plaque and the other being psoriasis-like papulovesicular and scaly lesions (16). In SCLE, interface dermatitis is usually severe, with many cellular bodies, and the lymphocyte infiltration is superficial, mainly around the blood vessels. The epidermis is thinner, keratinization is excessive, there are follicular plugs, mucin deposition, and basement membrane thickening less prominent than in discoid LE (18). Approximately one-third of SCLE cases are drug-induced, most commonly involving hydrochlorothiazide, calcium channel blockers, angiotensin-converting enzyme inhibitors, proton pump inhibitors, terbinafine, anti-TNF drugs, and anticonvulsants, among others (19). Recent literature reviews have revealed that anti-TNF drugs, proton pump inhibitors, and anticancer drugs, especially immune checkpoint inhibitors, are emerging as inducers of CLE. Subacute cutaneous lupus erythematosus is a rare but known adverse event during treatment with other anti-PD-1 antibodies such as pembrolizumab or nivolumab (20–24). To date, no cases of SCLE induced by Camrelizumab treatment have been reported, and we describe for the first time the occurrence of SCLE in patients receiving Camrelizumab treatment, confirmed by histopathology and serology.

## Case description

**Figure 1:**
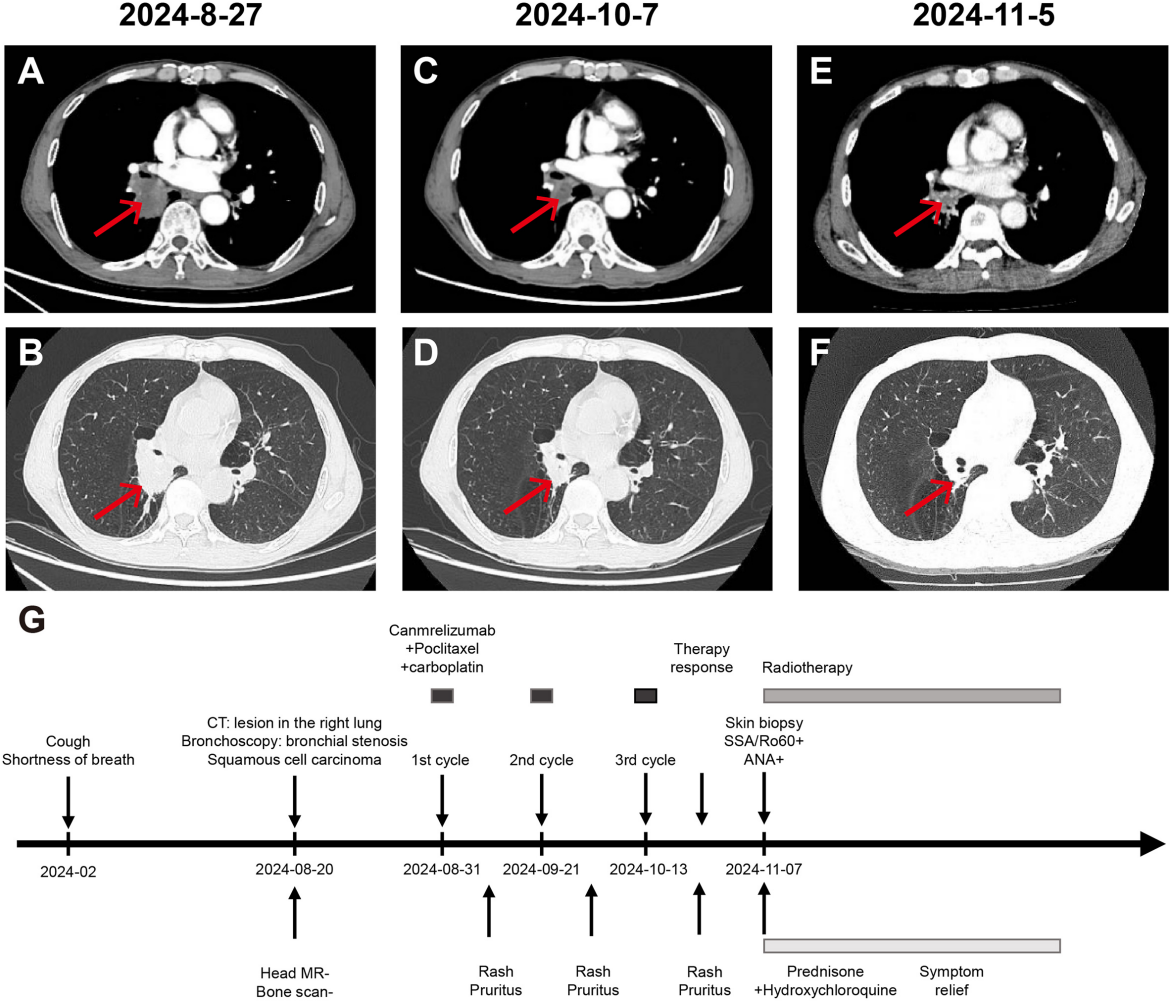
Chest CT assessment of the patient’s treatment progress and treatment effect. **(A, B)** Enhanced chest and abdomen CT on 27th August 2024 shows: 1. Central type lung cancer in the lower lobe of the right lung invading the middle lobe and lower lobe bronchus, segmental atelectasis and obstructive pneumonia in the lower lobe. 2. Pulmonary emphysema changes in both lungs. 3. Enlarged lymph nodes in the right hilum, suggesting a high likelihood of metastatic tumor. **(C, D)** Enhanced chest and abdomen CT on 7th October 2024 shows: 1. After lung cancer treatment, the lower lobe of the right lung posterior segment is smaller than before, invading the middle lobe and lower lobe bronchus, segmental atelectasis and obstructive pneumonia, with mucus plugs in the bronchi, showing improvement compared to before. 2. Enlarged lymph nodes in the right hilum, suggesting a high likelihood of metastatic tumor, similar to before. **(E, F)** Enhanced chest and abdomen CT on 5th November 2024 shows: 1. After lung cancer treatment, a cancer lesion adjacent to the hilum of the right lung lower lobe, slightly smaller than before, accompanied by segmental atelectasis and obstructive pneumonia in the lower lobe, with mucus plugs in the bronchi, showing slight improvement compared to before. 2. Enlarged lymph nodes in the right hilum, suggesting a high likelihood of metastatic tumor, similar to before. **(G)** Time line of the patient’s treatment course.

The patient is a 60-year-old male who presented with cough, production of white foamy sputum, and dyspnea with exertion 1 year prior. The cough increased in frequency and severity with exertion 6 months prior. There was no chest tightness, chest pain, fever, or hemoptysis. In August 2024, a chest CT scan revealed a lesion in the posterior segment of the lower lobe of the right lung, which was considered a central type lung cancer with obstructive pneumonia (**Figures 1A, B**). On August 28, 2024, a bronchoscopy showed that the right lower lobe bronchus was significantly narrowed, with extensive necrotic material covering it. The other lobar bronchi were patent, with mild erythema of the mucosa. No new growth or narrowing was seen, and no active bleeding was noted. Histopathological examination showed (right main bronchus mucosa) squamous cell carcinoma (middle-low differentiation). The immunohistochemistry results were as follows: CK7 (-), Ki-67 (20%+), Syn (-), TTF-1 (-), P40 (+), P63 (+), CK5/6 (+). Head MR and bone scan showed no significant abnormalities. The diagnosis was lung malignancy (squamous cell carcinoma cT2N1M0 IIb stage) and chronic obstructive pulmonary disease. The thoracic surgery department was consulted and advised against surgery. The patient underwent three cycles of anti-tumor therapy administered every 3 weeks (August 31, September 21, and October 13, 2024) with the following regimen: Paclitaxel 210 mg (Day 1), Carboplatin 340 mg (Day 1), Camrelizumab 200 mg (Day 1, intravenous infusion). The first course of treatment resulted in the appearance of multiple small papular lesions on the upper extremities, with significant itching, which progressively coalesced into patches and involved the face, neck, trunk, and upper extremities. Recurrent oral ulcers were also noted. No significant joint pain was noted, and the patient was considered to have eczema, which was treated symptomatically without improvement second and third courses of treatment resulted in a worsening of the rash and unbearable itching. Before receiving Camrelizumab, he had never experienced a rash, and had no family history of SLE or other autoimmune diseases.

**Figure 2:**
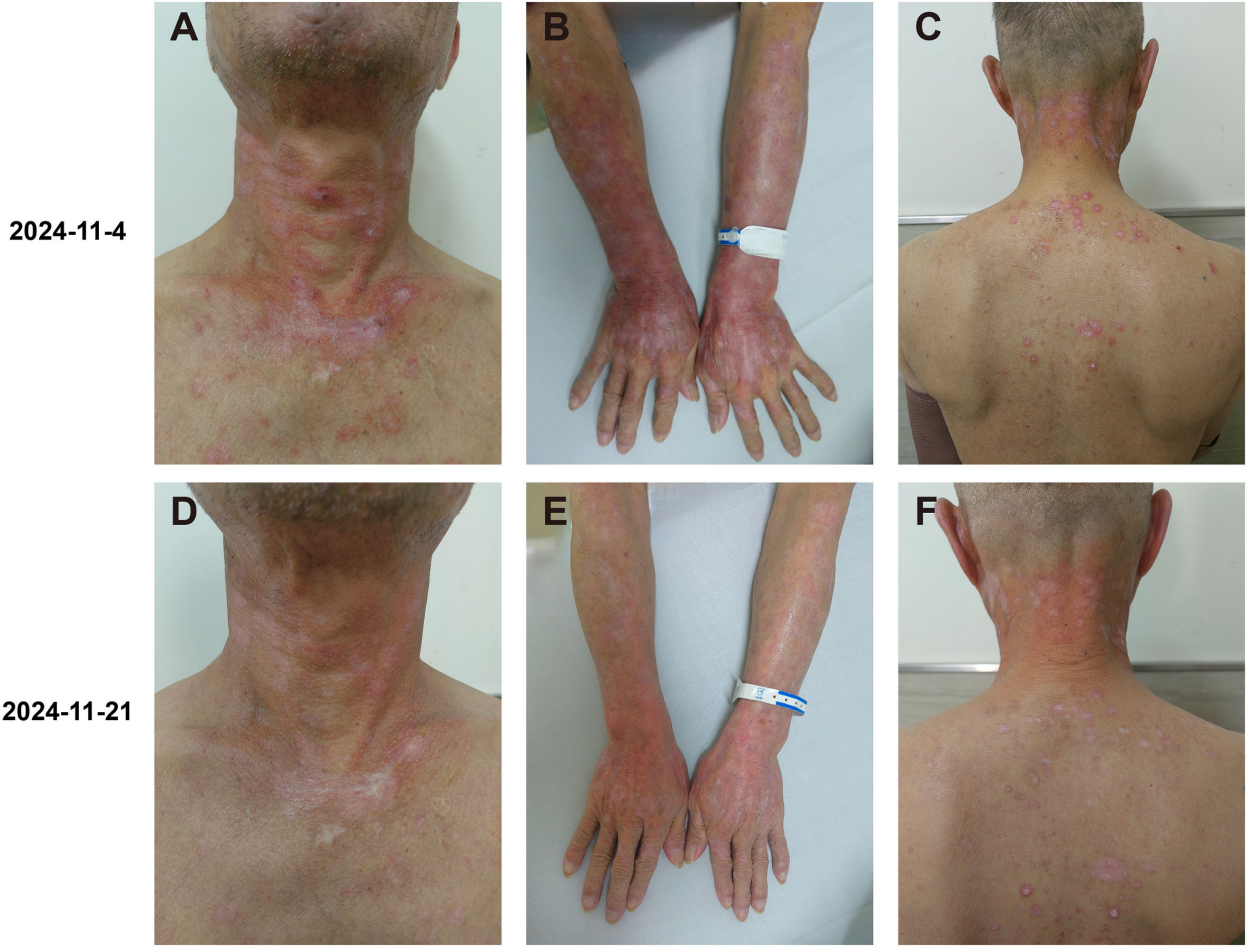
Clinical images of the patient’s rash. **(A–C)** At the first visit, the sun-exposed skin (on the neck and back, the forearm, and the back of the hand) presented severe itching, scales, bright red patches, red papules, and maculopapules. There was mild edema in multiple areas on the face, neck, and both upper limbs, with a sense of infiltration. **(D–F)** Fourteen days after the treatment with glucocorticoids and hydroxychloroquine was started, the itching and rash improved significantly.

**Figure 3:**
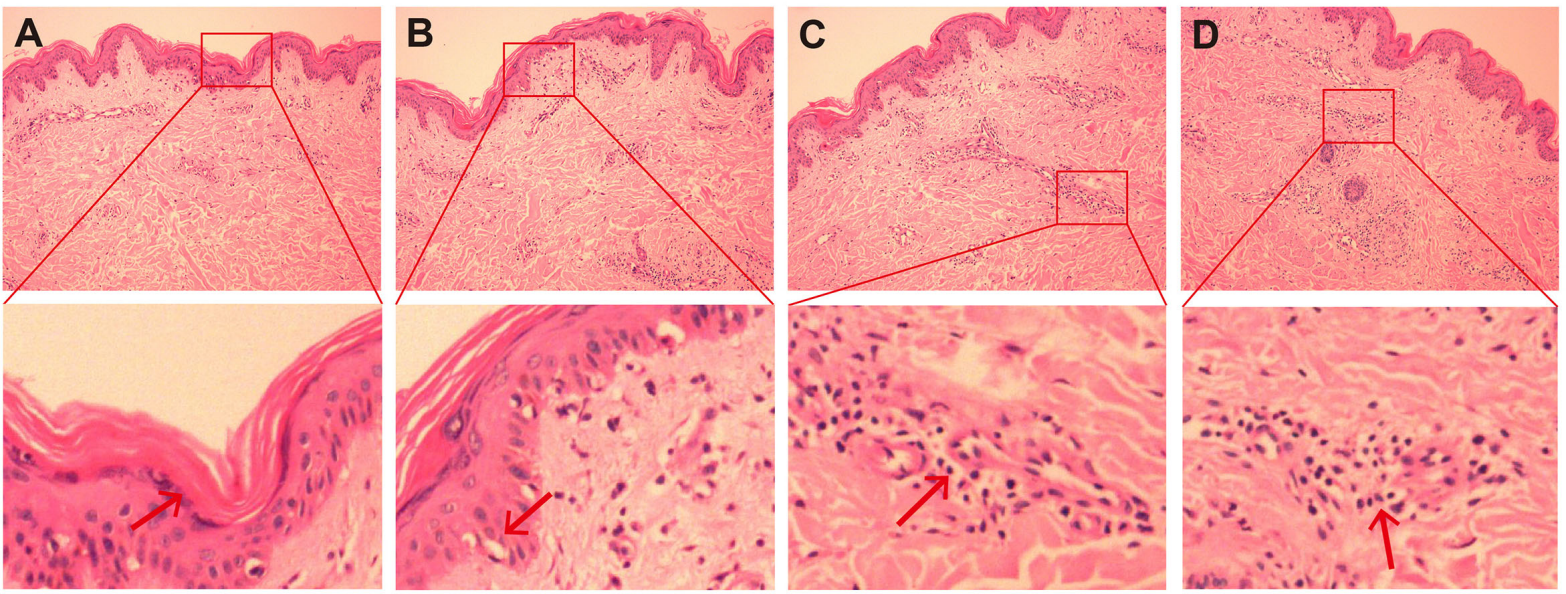
HE staining image of skin biopsy. **(A–D)** HE staining images of the skin on the right upper arm (original magnification, ×200). The submitted skin tissue was 0.7 × 0.3 × 0.2 cm in size. There was hyperkeratosis, and the epidermis was basically normal **(A)**. The melanin granules in the basal layer increased, and there were scattered liquefactive degeneration in the basal layer **(B)**, a small amount of pigment incontinence. In the superficial and middle layers of the dermis, there were scattered lymphocyte-dominated inflammatory cell infiltrations around blood vessels and appendages **(C, D)**, and a small number of plasma cells could be seen. Scattered individual lymphocytes and plasma cells could also be seen in the collagen.

On November 4, 2024, the patient presented to our clinic for consultation. Physical examination revealed multiple red plaques on the face, neck, and upper limbs, slightly swollen with induration, and scaly skin on the back, involving the periungual area, xerosis, and hypopigmentation on the trunk, multiple red papules and macules on the trunk (**Figures 2A–C**). Notably, the patient exhibited no clinical manifestations of oral ulcers, alopecia, arthralgia, or serositis (including pleuritis or pericarditis). On November 7, 2024, the anti-SSA/Ro60KD antibody was weakly positive (↑); the results of the two autoimmune antibody tests were as follows: anti-dsDNA antibody 18.582IU/ml; ANA1:320 positive (↑); ANA nuclear pattern 1 granular type. Anti-dsDNA antibody, anti-SmD1 antibody, anti-SSA/Ro52KD antibody, anti-SSB/La antibody, histone, etc. were all negative; renal function and muscle enzymes were normal. The skin biopsy of the right upper arm showed: the submitted skin tissue had keratinization excess, normal epidermis, increased melanin granules in the basal layer, scattered liquefaction degeneration in the basal layer, a few dyschromatophilia, mild perivascular and perivascular lymphocyte-predominant inflammatory cell infiltration, and scattered individual lymphocytes and plasma cells in the dermis (**Figures 3A–D**). The patient was diagnosed with drug-induced immune-mediated SCLE, CTCAE v5.0 grade 2 adverse event. According to the opinion of the dermatology specialist, the patient was given photoprotective measures. Considering the extensive rash and significant pruritus that affected the patient’s life, local corticosteroids and a small dose of prednisone 20mg qd were administered orally for 2-4 weeks, then gradually reduced to a maintenance dose of 5mg of prednisone. The total treatment course was half a year, and hydroxychloroquine 0.2g bid was also given orally. The patient’s pruritus was significantly relieved after about 5 days of medication, and the rash decreased after 2 weeks (**Figures 2D–F**). The patient was followed up in the dermatology outpatient department. The patient’s imaging examination showed a treatment response (**Figures 1C–F**). As surgery was not feasible, the patient subsequently received radical radiotherapy. After discussing the risks and benefits of continued treatment with the patient, both parties decided to discontinue Camrelizumab treatment and actively monitor the cancer. The patient’s treatment process is detailed in the flowchart (**Figure 1G**).

## Discussion

Immune checkpoint inhibitors have achieved significant breakthroughs in the treatment of lung cancer, bringing significant survival benefits to patients. However, irAEs occur from time to time, and the specific mechanism behind their occurrence is still unclear. It is generally believed that excessive immune responses may be caused by autoimmune T cells, thereby triggering symptoms in relevant organs. We herein report a case of SCLE induced by Camrelizumab treatment. The patient’s rash appeared and gradually worsened after one cycle of ICI treatment, presenting as coexisting multiple annular plaques and scaly lesions (25). The differential diagnosis of CLE varies depending on the appearance of the lesion and the possible presence of other systemic symptoms. In the annular lesions of SCLE, annular granuloma, erythema annulare centrifugum, and erythema gyratum repens should be considered. For the papulosquamous rash of SCLE, the differential diagnosis includes psoriasis and its photosensitive variant or photoallergic drug eruption (26). The patient had no other systemic symptoms, including arthritis/arthralgia, discomfort, and myalgia, nor did he have internal organ involvement such as renal or neurological diseases. Consistent with the description by Qianjin Lu et al. that 70% of SCLE patients are positive for Ro(SSA) and 70-80% are positive for ANA, the patient in this case was positive for both Ro(SSA) and ANA. The pathological findings of the patient were consistent with interfacial dermatitis, featuring epidermal thinning, hyperkeratosis, lymphocyte infiltration, and were also consistent with the description by Blake SC et al. (25). The patient only had cutaneous lupus erythematosus manifestations and did not meet the criteria for SLE.

In a report of 472 patients by Bataille P et al., 109 drugs that induce CDILE were identified, including anti-TNFα, proton pump inhibitors, anti-cancer drugs, especially checkpoint inhibitors, as emerging drugs in CDILE (26). Some case reports have indicated that ICI therapy is also associated with SCLE. Fietz S et al. reported a case of a metastatic squamous cell carcinoma patient who developed positive anti-SS-A/Ro antibodies after receiving Cemiplimab treatment and was diagnosed with SCLE and immune-related hepatitis (21).In a large retrospective study of 4,487 cancer patients, 8 cases of ICI-induced SCLE were reported, with 2 cases attributed to Nivolumab (27). Cases of SCLE induced by PD-1 inhibitor Pembrolizumab, PD-L1 inhibitor Atezolizumab and Durvalumab, and CTLA-4 inhibitor Ipilimumab have also been reported in the literature (28–31). Not only anti-PD-1 inhibitors, but also PD-L1 inhibitors and CTLA-4 inhibitors may induce SCLE, with anti-PD-1 inhibitors being more frequently reported. This patient developed SCLE after anti-PD-1 treatment. There are also reports of paclitaxel, an anti-tumor drug, causing SCLE (32–34). This patient received paclitaxel combined with carboplatin chemotherapy while using Camrelizumab. Although SCLE caused by chemotherapy drugs is not common, it cannot be completely ruled out that other drugs or factors besides Camrelizumab may have caused SCLE.

A multidisciplinary team consisting of dermatologists, rheumatologists, nephrologists, and general practitioners is key to the optimal management of patients with systemic symptoms. Patients with only skin manifestations can be treated and monitored by dermatologists (26). Photoprotection is the basic pillar of SCLE treatment, and corticosteroids are considered the first-line local treatment due to their anti-inflammatory effects. Antimalarial drugs (AM) are the first-line systemic treatment and may prevent CLE from progressing to systemic disease (35). Hydroxychloroquine (HCQ) is the most commonly used adjunctive therapy, as it has better ocular safety compared to chloroquine (CQ), but still needs to be monitored for the most relevant side effect of retinal changes, which occurs in up to 1% of cases (36). Alternative systemic treatments include methotrexate, oral retinoic acid, aminopyrine, and thalidomide, among others. As our understanding of the disease pathogenesis progresses, new treatment strategies have been developed targeting the identified different immune activation pathways (16). In this case, the patient’s pruritus symptoms improved significantly after discontinuation of ICI therapy and treatment with prednisone and hydroxychloroquine, and the rash gradually faded. As the treatment course needs to be more than half a year, the side effects of corticosteroids and antimalarial drugs need to be closely monitored. As of the time of reporting, the patient had no adverse reactions to corticosteroids or antimalarial drugs.

The exact pathogenesis of SCLE is unknown, but it is believed to be multifactorial. Research into the genetic factors involved in CLE is still in its infancy, and UVR is the most clearly identified trigger factor for CLE, with other factors including keratinocyte apoptosis, activation of the innate immune system to promote tissue inflammation, and elevated levels of IFN, among others (37). The pathogenesis of ICI-related SCLE remains unclear, and several possible theories have been proposed. Firstly, SCLE may be stimulated by UV-B radiation, leading to Ro (SSA) antigen translocation and increased cell membrane antigen expression through epitope spreading (38). Then, anti-PD-1 or PD-L1 may regulate humoral immunity, thereby enhancing pre-existing autoantibodies and revealing underlying autoimmunity (39). Therefore, anti-PD-1 treatment may cause SCLE by increasing the immune system’s activity against antigens in both cancer and healthy tissues, as well as by raising the levels of pre-existing autoantibodies. The patient did not receive a timely diagnosis of SCLE after the onset of the rash, and clinicians lack awareness of this disease, which needs to be addressed. Up to 5% to 25% of isolated CLE cases, regardless of subtype, may progress to SLE during the course of the disease (40), and therefore, regular follow-up with shorter intervals is recommended, along with comprehensive clinical examination and thorough laboratory investigation. Unlike classic drug-induced SCLE, patients may develop multiple autoimmune diseases, including autoimmune hepatitis and myositis, among others (20, 21), and subsequent treatment should be cautious when challenging immune checkpoint inhibitors. After discussing the risks and benefits of continued treatment with this patient, we both decided to discontinue Camrelizumab treatment and actively monitor the cancer.

This study has several limitations that should be acknowledged. First, the absence of direct immunofluorescence (DIF) testing on pathological specimens precluded comprehensive evaluation of immunoglobulin (IgG, IgM) and complement component (C3) deposition patterns at the dermal-epidermal junction, which could have provided valuable diagnostic information. Second, while we observed potential associations between PD-1 inhibitor use and subacute cutaneous lupus erythematosus (SCLE) development, the critical pharmacological determinants remain undefined. The structural heterogeneity among various PD-1 inhibitor agents - particularly variations in molecular conformation and binding epitopes may differentially influence autoimmune pathway activation, but this hypothesis requires systematic validation through comparative molecular studies.

## Conclusion

This case report describes a 60-year-old male patient with lung squamous cell carcinoma and ICI-induced SCLE, which was successfully treated with local and systemic steroids and hydroxychloroquine, leading to rapid resolution of the rash. Early recognition and management of ICI-induced SCLE are crucial for the continuity of cancer treatment, subsequent drug selection, and improvement of patient outcomes.

## Data Availability

The original contributions presented in the study are included in the article/supplementary material. Further inquiries can be directed to the corresponding authors.
